# Functional consequences of piceatannol binding to glyceraldehyde-3-phosphate dehydrogenase

**DOI:** 10.1371/journal.pone.0190656

**Published:** 2018-01-03

**Authors:** Joanna Gerszon, Eligiusz Serafin, Adam Buczkowski, Sylwia Michlewska, Jakub Antoni Bielnicki, Aleksandra Rodacka

**Affiliations:** 1 Department of Molecular Biophysics, Faculty of Biology and Environmental Protection, University of Lodz, Lodz, Poland; 2 Bionanopark Ltd., Lodz, Poland; 3 Laboratory of Computer and Analytical Techniques, Faculty of Biology and Environmental Protection, University of Lodz, Lodz, Poland; 4 Unit of Biophysical Chemistry, Department of Physical Chemistry, Faculty of Chemistry, University of Lodz, Lodz, Poland; 5 Department of General Biophysics, Faculty of Biology and Environmental Protection, University of Lodz, Lodz, Poland; 6 Laboratory of Microscopic Imaging and Specialized Biological Techniques, Faculty of Biology and Environmental Protection, University of Lodz, Lodz, Poland; Russian Academy of Medical Sciences, RUSSIAN FEDERATION

## Abstract

Glyceraldehyde-3-phosphate dehydrogenase (GAPDH) is one of the key redox-sensitive proteins whose activity is largely affected by oxidative modifications at its highly reactive cysteine residue in the enzyme’s active site (Cys149). Prolonged exposure to oxidative stress may cause, *inter alia*, the formation of intermolecular disulfide bonds leading to accumulation of GAPDH aggregates and ultimately to cell death. Recently these anomalies have been linked with the pathogenesis of Alzheimer’s disease. Novel evidences indicate that low molecular compounds may be effective inhibitors potentially preventing the GAPDH translocation to the nucleus, and inhibiting or slowing down its aggregation and oligomerization. Therefore, we decided to establish the ability of naturally occurring compound, piceatannol, to interact with GAPDH and to reveal its effect on functional properties and selected parameters of the dehydrogenase structure. The obtained data revealed that piceatannol binds to GAPDH. The ITC analysis indicated that one molecule of the tetrameric enzyme may bind up to 8 molecules of polyphenol (7.3 ± 0.9). Potential binding sites of piceatannol to the GAPDH molecule were analyzed using the Ligand Fit algorithm. Conducted analysis detected 11 ligand binding positions. We indicated that piceatannol decreases GAPDH activity. Detailed analysis allowed us to presume that this effect is due to piceatannol ability to assemble a covalent binding with nucleophilic cysteine residue (Cys149) which is directly involved in the catalytic reaction. Consequently, our studies strongly indicate that piceatannol would be an exceptional inhibitor thanks to its ability to break the aforementioned pathologic disulfide linkage, and therefore to inhibit GAPDH aggregation. We demonstrated that by binding with GAPDH piceatannol blocks cysteine residue and counteracts its oxidative modifications, that induce oligomerization and GAPDH aggregation.

## Introduction

Many different hypotheses attempting to explain the formation of changes leading to the development of Alzheimer's Disease (AD) have appeared in recent years. Numerous studies have already established Aβ amyloidogenesis as a hallmark of AD pathogenesis [1–3]. It has been shown that glyceraldehyde-3-phosphate dehydrogenase (GAPDH) plays a substantial role in this processes. GAPDH is a ubiquitous enzyme (constituting ~10–20% of the total cellular protein content) that catalyzes the key step of glycolysis [4]. It is now well established that besides the traditional role in a glucose metabolism, GAPDH is implicated in the regulation of cell proliferation, DNA repair, tRNA export, membrane fusion and transport, cytoskeletal dynamics, cell death and many other cellular processes [5]. Due to the presence of a reactive, nucleophylic cysteine residue in the active site (Cys149), this multifunctional enzyme is particularly susceptible to oxidative modifications not only by reactive oxygen and nitrogen species, but also by peptide and protein peroxides and hydroperoxides [6–10]. It has been demonstrated that this cysteine, to the largest extent, is involved in the formation of aggregates and oligomers during prolonged exposure of GAPDH to oxidative stress [11–14]. Further, Takeuchi's team [11–14] indicated that GAPDH in aggregated form exhibits significant functional similarity to aggregates formed by the amyloid beta peptide. Both *in vitro* and *in vivo* studies have also demonstrated that GAPDH aggregates accelerate Aβ amyloidogenesis and contribute to neuronal cell death [13]. GAPDH has also been shown to be one of the major components of amyloid deposits, neurofibrillary tangles, senile plaques and Lewy bodies in post mortem brain tissues derived from patients with diagnosed Alzheimer's and Parkinson's disease. The presented data confirm the significant contribution of oxidative-modified GAPDH in the pathogenesis of Alzheimer's disease [11–14].

In order to further explain the importance of glyceraldehyde-3-phosphate dehydrogenase in the development of neurodegenerative diseases, it would be helpful to identify effective inhibitors that would prevent the GAPDH translocation to the nucleus, and inhibit or slow down its aggregation and oligomerization. Up to now, the data about chemicals able to interact with GAPDH or influence its aggregation processes are insufficient. However, the novel evidence indicate that low molecular compounds may have beneficial effect in that regard [15]. It is therefore reasonable to examine naturally occurring compounds, with low molecular weight that are present in our daily diet, which are non-toxic and exhibit pro-health properties for neuronal cells. Stilbene derivatives are of particular interest for the neuroprotection research area. Its neuroprotective properties include antioxidant, anti-inflammatory, anti-apoptotic, as well as anti-amyloidogenic activities [16–19]. There are some evidences which indicate that the long term treatment with resveratrol, one of the most common polyphenols, promotes neuroprotection [19–22]. Nonetheless the rapid metabolism and low bioavailability of resveratrol limits its application [23–25]. Therefore, it is very important to examine the effects of its metabolites on cells. Both *in vitro* and *in vivo* assays revealed differences in hydrophilicity/lipophilicity of the polyphenolic compounds caused by the metabolic modifications, such as the addition of hydroxyl, glucuronyl or sulfate groups. These modifications are predicted to have a huge impact on the compound’s biochemical activities for example due to the differences in the cellular membrane permeability. For example one of the major resveratrol’s metabolite is piceatannol, its hydroxyl analogue, also found in large quantities in daily diet products (fruits, vegetables). This compound fulfills the conditions of exceptional neuroprotector, since it has high antioxidant potential, the ability to cross the blood-brain barrier, the ability to inhibit inflammation processes and prevent excessive protein aggregation [24]. We have previously demonstrated that piceatannol have beneficial effect on neuronal cells [17]. It enhances the activity of glutathione peroxidase (GPx) enzymes and increases the expression of GPx. Besides, piceatannol promotes neuronal cell survival by attenuation of oxidative stress-mediated cell damage and by sustaining sirtuin 1 (SIRT1), brain derived neurotrophic factor (BDNF), and seladin-1 mRNA on constant level [17].

Therefore, the main aim of this research was to establish if piceatannol binds to GAPDH and whether this interaction affects protein’s functional properties and inhibits GAPDH aggregation.

## Materials and methods

### Materials

Glyceraldehyde-3-phosphate dehydrogenase (GAPDH) from rabbit muscle, trans-3,5,3’,4’-tetrahydroxistilbene (piceatannol, PIC), trans-3,5,4′-trihydroxystilbene (resveratrol, RSV) and all other chemicals were purchased from Sigma-Aldrich chemical Co (St. Louis, MO, USA), unless otherwise stated.

### Preparation of protein and polyphenol solutions

Protein solution was prepared by dissolving GAPDH in 0.02 M sodium phosphate buffer at pH 7.4. Protein concentration was determined spectrophotometrically at 280 nm, using the extinction coefficient E_1%_ = 10 (GAPDH, MW = 143,000). The stock solutions of piceatannol and resveratrol were freshly prepared by dissolving the commercial powders in phosphate buffer, in dark. The molar extinction coefficient for piceatannol was 33100 M^-1^cm^-1^ at the maximal wavelength of 326 nm [26], and 30335 M^-1^cm^-1^ for resveratrol at the wavelength of 304 nm [27].

### Assay of GAPDH activity in the presence of piceatannol

The activity of the GAPDH (2 μM) and LDH (2 μM) was determined spectrophotometrically by measuring the rate of reduction of NAD^+^ to NADH (in case of GAPDH) or oxidation of NADH to NAD^+^ (for LDH), what has been described in details previously [10]. The relative activity of enzymes incubating with various concentrations of piceatannol (0; 3.2; 16; 32; 64 μM) was expressed as a percentage of the activity of control (enzyme without piceatannol) and it was determined during 120 min of incubation. Since the effect of piceatannol on LDH activity was not observed, in the figure there are presented data only for LDH treated with the highest concentration of compound (64 μM). All measurements were carried out at 24 ± 1°C using a CARY-1 apparatus (Varian, Melbourne, Australia).

### Detection of GAPDH thiols during incubation with piceatannol

Detection of relative content of protein SH groups was based on the formation of color product resulting from reaction of DTNB (Ellman’s reagent). Absorbance at 412 nm was measured spectrophotometrically in denaturation condition (4 M guanidine-HCl). Relative changes in the content of free SH groups in GAPDH exposed on piceatannol was presented as relative intensity of absorbance.

### Isothermal titration calorimetry

The thermal effects of titration of aqueous GAPDH solution (5 μM) with aqueous piceatannol solution (0.5 mM) were determined at 25°C using the isothermal titration calorimetry (VP-ITC calorimeter, MicroCal). A piceatannol solution volume of 5 μl from the syringe was added every 1000 seconds to the dehydrogenase solution in the ITC cell. Single injection time was 10 seconds. The thermal effects of dilution of the piceatannol solution were determined independently by adding the compound to water in portions, while keeping the calorimeter working parameters the same as during the titration. The thermal effects of the GAPDH—piceatannol direct interactions were calculated by subtracting the thermal effects of dilution of ligand solution from the corresponding thermal effects of protein solution titrated with the ligand solution. The determined binding isotherm may be described by the model of One Set of Sites using the non-linear multi-parameter regression (Origin MicroCal 7.0), according to Eq [1]:

$$Q=\frac{nM_{t}\Delta HV_{0}}{2}\left[1+\frac{X_{t}}{nM_{t}}+\frac{1}{nKM_{t}}-\sqrt{{\left(1+\frac{X_{t}}{nM_{t}}+\frac{1}{nKM_{t}}\right)}^{2}-\frac{4X_{t}}{nM_{t}}}\right]$$ (1)

where *M*_*t*_ and *X*_*t*_–GAPDH and piceatannol concentrations, *V*_0_–cell working volume (287.37 μl), *n*–stoichiometric parameter describing the number of protein binding centers (binding sites), *K*–binding constants between ligand and GAPDH binding center, Δ*H* and Δ*S*–molar enthalpy and entropy of binding between ligand and GAPDH binding center. The free enthalpy of binding was calculated using the basic thermodynamic relationships:

ΔG=ΔH−TΔS=−RTlnK (2)

### Zeta potential measurements

Zeta potential measurements were performed using a Zetasizer Nano ZS from Malvern, which employs electrophoretic light scattering techniques. The zeta potential was calculated directly from the Helmholtz−Smoluchowski equation using the Malvern software. Measurements of the potential were performed at 37°C with three repetitions. Increasing concentrations of polyphenol were added to 2 μM GAPDH and the zeta potential was measured.

### Molecular modeling and docking

The 3D structure of piceatannol was downloaded from PubChem (CID 667639). Docking studies were conducted using a BIOVIA Discovery Studio (Dassault Systems) molecular simulation system with the LigandFit and CDOCKER algorithms. Molecular graphics were produced using the UCSF Chimera package [28]. The GAPDH structure used in this work was from Protein Data Bank, obtained by X-ray diffraction (PDB 1J0X). The four monomers forming the tetramer are not 3D identical. Ligand poses are evaluated and prioritized according to the value of the dock-score function (D-S).

### Circular dichroism

The CD spectrum of GAPDH (2 μM) exposed to piceatannol (32 μM) for 50 and 100 min. was measured with a spectropolarimeter model J-815 (Jasco, Japan). The path length of the optical quartz cuvette was 5 mm for far-UV CD measurements at 195–250 nm. Before measurements samples of GAPDH were diluted in 0.01 M sodium phosphate buffer (pH 7.4) at a concentration of 0.28 μM. Spectra were obtained as the average of 3 successive scans with a bandwidth of 2 nm. The data were presented as molar residue ellipticity.

### Thioflavin-T binding-dependent fluorescence

To measure the aggregation of GAPDH thioflavin-T (ThT) assays were performed according to a previous report [11] with minor modifications. Thioflavin-T (100 μM) was dissolved in 50 mM glycine buffer, pH 8 and kept in the dark. GAPDH (4 μM) was incubated with piceatannol (50 μM) or resveratrol (50 μM) or with H_2_O_2_ (3 mM). There were also samples where GAPDH was preincubated for 30 minutes with piceatannol or resveratrol and afterwards hydrogen peroxide was added. All samples were incubated at 37°C. Twenty μl of GAPDH sample was mixed with 180 μl of thioflavin-T solution and the fluorescence intensity was measured after 3 min in a 96-well plate reader using Varian Cary Eclipse fluorimeter with microplate reader accessory. The fluorescence intensity was measured at an excitation wavelength of 450 nm and an emission of 482 nm.

### Congo Red Birefringence

Congo red staining was conducted using the procedure described by Nakajima et al. [11]. Aliquots (100 μl) of GAPDH (4 μM), as is or pretreated with piceatannol (50 μM) or resveratrol (50 μM) for 30 minutes and afterwards treated with hydrogen peroxide (3 mM) for 24 hours at 37°C were added to 900 μl of Congo Red solution (25 μg/ml in PBS). This mixture was incubated for 30 min at room temperature and then centrifuged at 15,000g for 30 min. The pellet was resuspended in 50 μl of Milli-Q water (Millipore). The drops of this suspension were allowed to dry on a slide glass for 10 min. The birefringence was determined in light microscope Nicon Eclipse 50i, using Coolview software.

### Transmission electron microscopy (TEM)

TEM assay was used to evaluate the changes of the structure of GAPDH after treatments with H_2_O_2_, piceatannol with H_2_O_2_ and resveratrol with H_2_O_2_. Ten microliters of every sample were placed on 200-mesh copper grids with carbon surface (Ted Pella). The samples were negatively stained with 2% uranyl acetate (Polysciences) for 40 seconds and dried at room temperature. The transmission electron microscopy images were created using JEOL-1010 (Japan) (TEM).

### Statistical analysis

Results are presented as mean ± SD. The statistical significance of the impact of concentration of piceatannol and incubation time was rated by two-way analysis of variance. For statistical evaluation of the effect of time of incubation on changes in the content of thiol groups was performed using one-way analysis of variance. STATISTICA, v. 13.0 was used for the calculations.

## Results

### The effect of piceatannol on the activity of GAPDH

**Fig 1:**
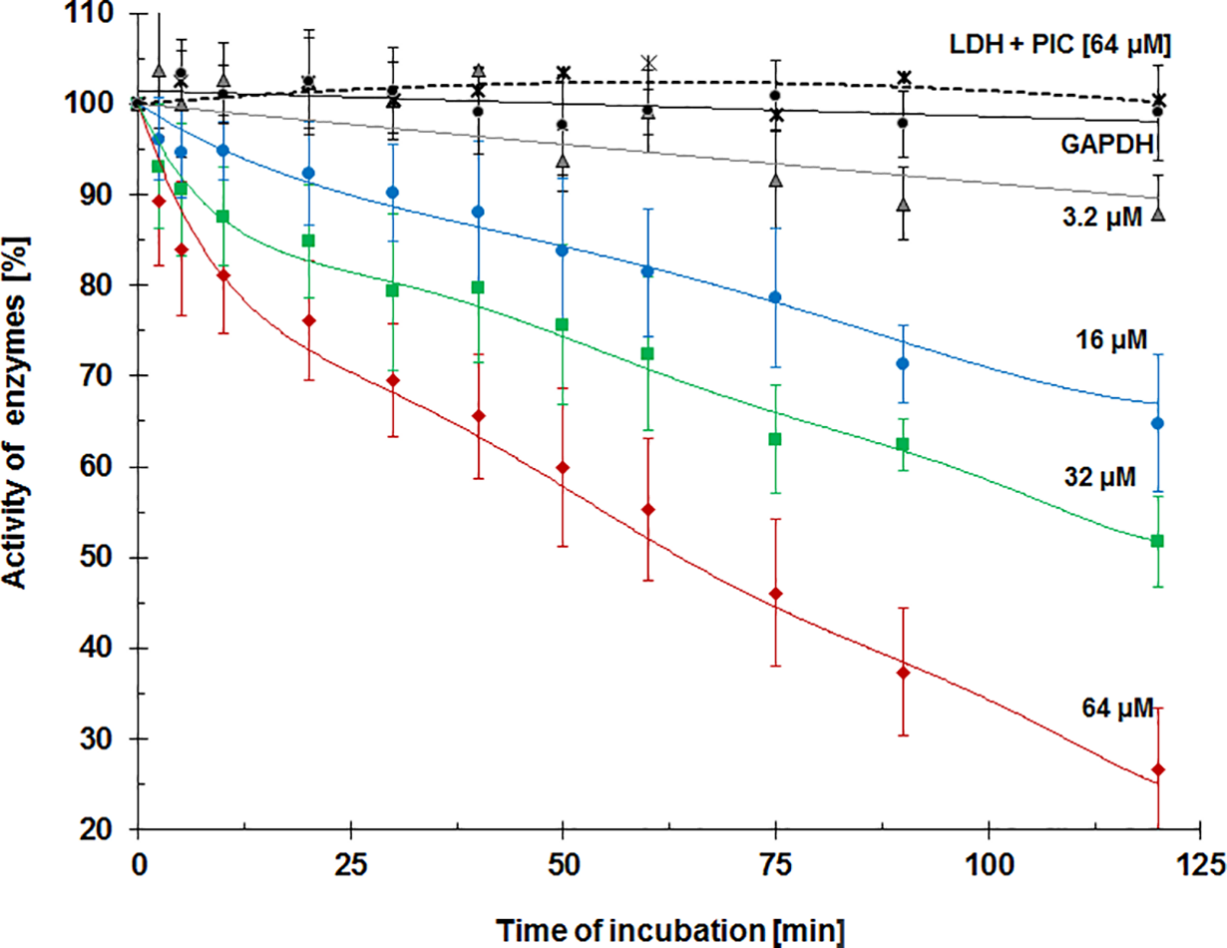
The effect of piceatannol on the activity of GAPDH, measured during 2 hours of incubation of GAPDH (2 μM) with piceatannol at concentrations 0, 3.2, 16, 32 and 64 μM (continuous lines). Similarly, the effect of piceatannol at the concentration 64 μM on the activity of LDH was determined (black dashed line). Catalytic activity of enzymes are expressed as percentages referring to the activity of untreated samples (control). Data are means ± SD of n = 4–8 independent measurements.

The activity of GAPDH treated with piceatannol was measured over 2 hours of incubation. It was shown that piceatannol in a concentration and time dependent manner declined GAPDH activity to a significant extent (Fig 1). The statistical analysis of changes in the GAPDH activity depending on the time and piceatannol concentration revealed a statistically significant effect of both factors, as well as their statistically significant interactions (p < 0.05).

**Fig 2:**
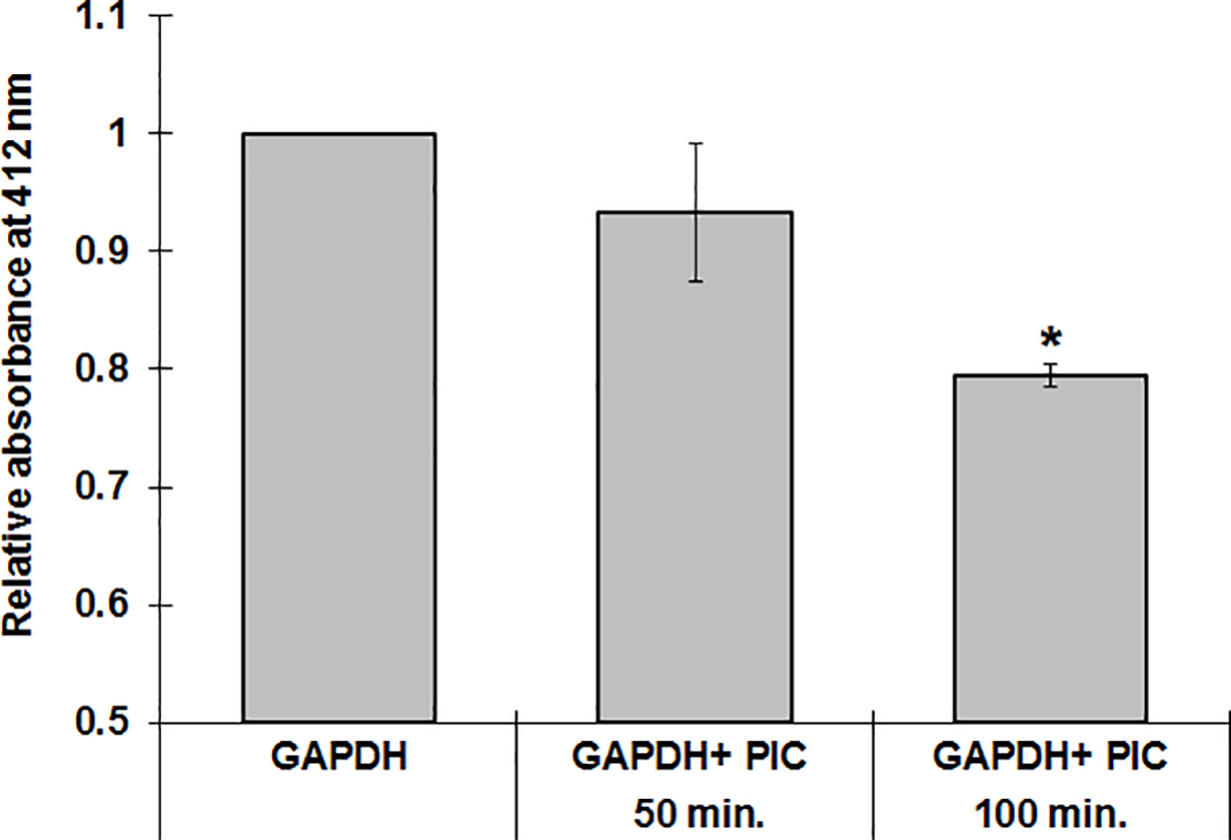
A decrease in the relative absorbance (λ = 412 nm) of the colored solution formed in the reaction of free SH groups present in GAPDH with DTNB. Data are means ± SD of n = 3–4 independent measurements, * p < 0.05.

Parallel with the enzyme inactivation a statistically significant decrease in the absorbance of the colored solution formed in the reaction of free SH groups with DTNB, was observed. This shows that piceatannol oxidizes or forms bonds with SH groups thereby blocking them from reaction with DTNB (Fig 2).

It is well known that GAPDH is thiol-dependent enzyme, which means that the catalytic activity depends on Cys149 residues (human analogues are Cys152) which are required for nucleophilic attack on the substrate glyceraldehyde 3-phosphate (G3P) (9). Cysteinyl sulfur reactivity is interdependent on pH and pKa. It was found that Cys152 is characterized by a low pKa 6.03, and at physiological pH, this sulfhydryl group exists in the highly reactive nucleophilic thiolate state (R‒S^‒^). Cys thiolates are considerably more nucleophilic than their protonated forms and can be more easily oxidized and/or formed adducts with catechol moiety which is present in stilbene derivative molecule (i.e. in piceatannol).

### Calorimetric (ITC) determination of protein-ligand binding parameters

**Fig 3:**
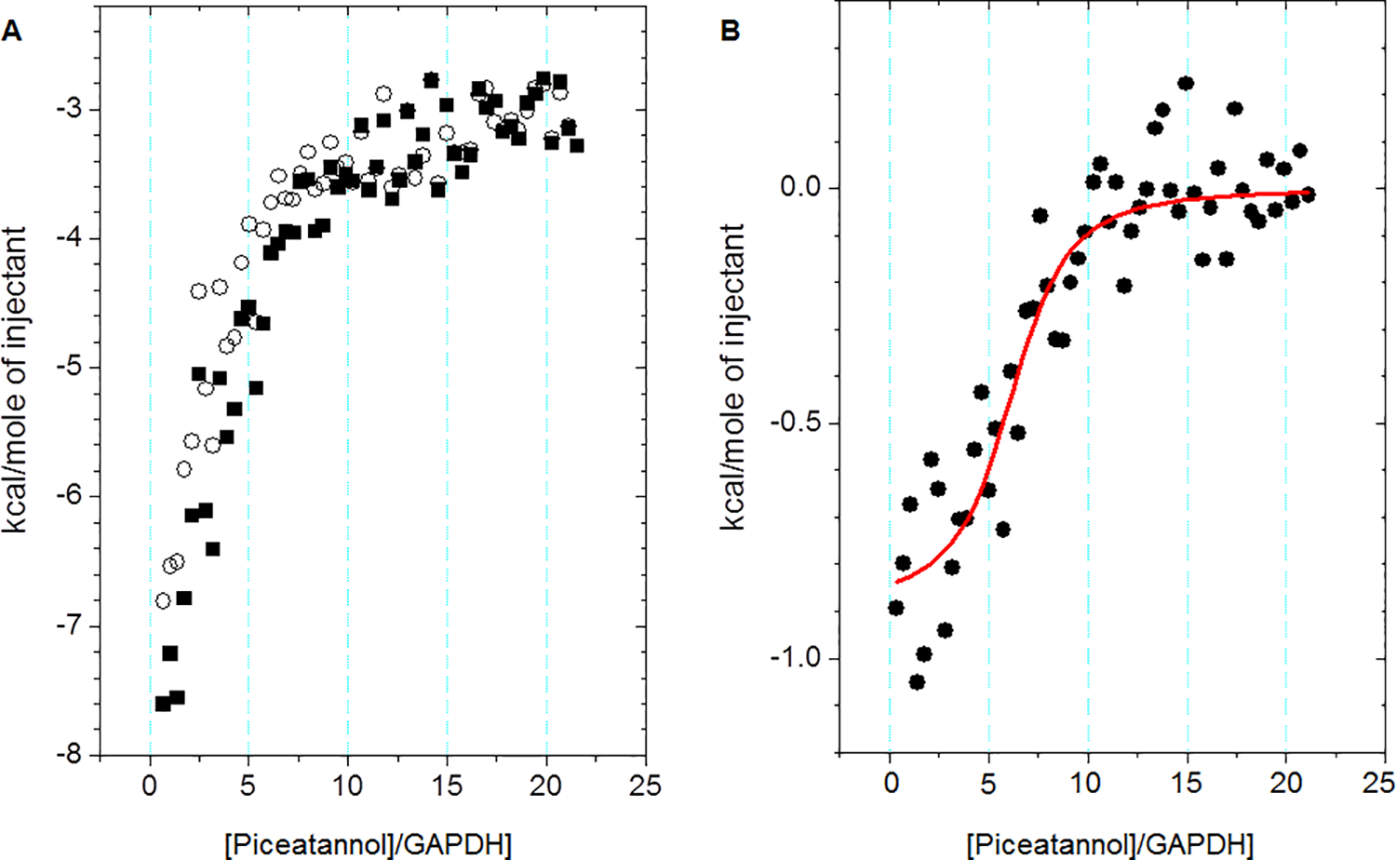
**(A)** Thermal effects of the titration 0.5 mM piceatannol solution (in the syringe) into 5 μM GAPDH protein solution (in the cell) (■) and corresponded to them thermal effects of dilution of the piceatannol in pure water (○). **(B)** Thermal effect of the interaction between protein GAPDH and piceatannol corrected with the piceatannol dilution effects and calculated per one mole of the ligand. Solid line is approximation of ITC date of GAPDH by piceatannol using the model of one set of binding sites.

The thermal effects of titration of the aqueous glyceraldehyde-3-phosphate dehydrogenase solution (5 μM) in the cell with the aqueous solution of piceatannol (0.5 mM) in the syringe was performed at 25°C using the isothermal titration calorimetry. The corresponding effects of piceatannol dilution in water were determined independently (Fig 3A). The dilution effects of GAPDH solution were negligible, as compared to the other measured effects. Subsequently, the effects associated with the ligand (piceatannol) dilution were subtracted from the thermal effects of GAPDH titration with the ligand. The resulting thermal effects of the direct GAPDH-piceatannol interaction are exothermic (Fig 3B).

Enthalpogram of these direct interactions of GAPDH with piceatannol (Fig 3B) was described by the model of One Set of Binding Sites (Eq 1). Non-linear multi-parameter regression was performed using Origin MicroCal 7.0 software. The determined parameters are summarized in Table 1.

**Table 1:** Binding parameters of the interaction of GAPDH protein with piceatannol in water solution at 25°C determined by isothermal titration calorimetry.

*n*	*K* [l mol^-1^]	Δ*H* [kcal mol^–1^]	Δ*S* [cal K^–1^ mol^–1^]	Δ*G* [kcal mol^–1^]
7.3 ± 0.9	57 000 ± 26 000	–0.886 ± 0.065	23.4 ± 1.1	–7.85 ± 0.28

The resulting stoichiometric parameter n (Table 1) indicates that, within the measurement uncertainty limits, GAPDH has up to eight piceatannol binding sites. The possibility of describing the piceatannol-GAPDH binding isotherm with the model of One Set of Sites suggests that all GAPDH binding sites exhibit similar affinity for the ligand (piceatannol). The binding of piceatannol by GAPDH is spontaneous (ΔG<0) and exothermic (ΔH<0), indicating that the effects of direct piceatannol-protein interaction dominate over the effects of partial dehydration of ligand and GAPDH functional groups. The entropy of binding (ΔS>0) indicates an increase in the degree of disorder of the reagents during saturation of GAPDH active sites with piceatannol. The high binding constant value of piceatannol to the GAPDH active site (logK>3) indicates that the supramolecular complex formed is relatively stable [29,30].

### Secondary structure changes in GAPDH exposed to piceatannol

**Fig 4:**
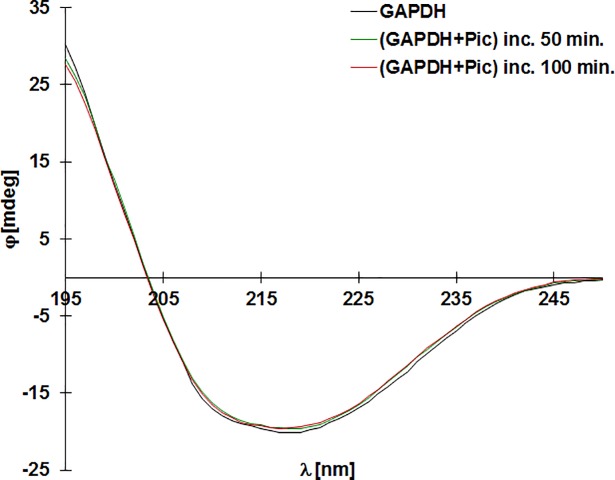
Far-UV CD spectra of native GAPDH and GAPDH treated with 32 μM of piceatannol at room temperature for 50 and 100 min of incubation. The data are expressed as molar residue ellipticity.

Despite substantial changes in the activity of GAPDH treated with piceatannol, no significant changes were observed in the secondary structure of the enzyme (Fig 4). The content of α-helix (30%), beta sheet (35%) and random coil (35%) remained unchanged even after 50 and 100 minutes of incubation with stilbene derivative at concentration 32 μM.

### Measurements of zeta potential

**Fig 5:**
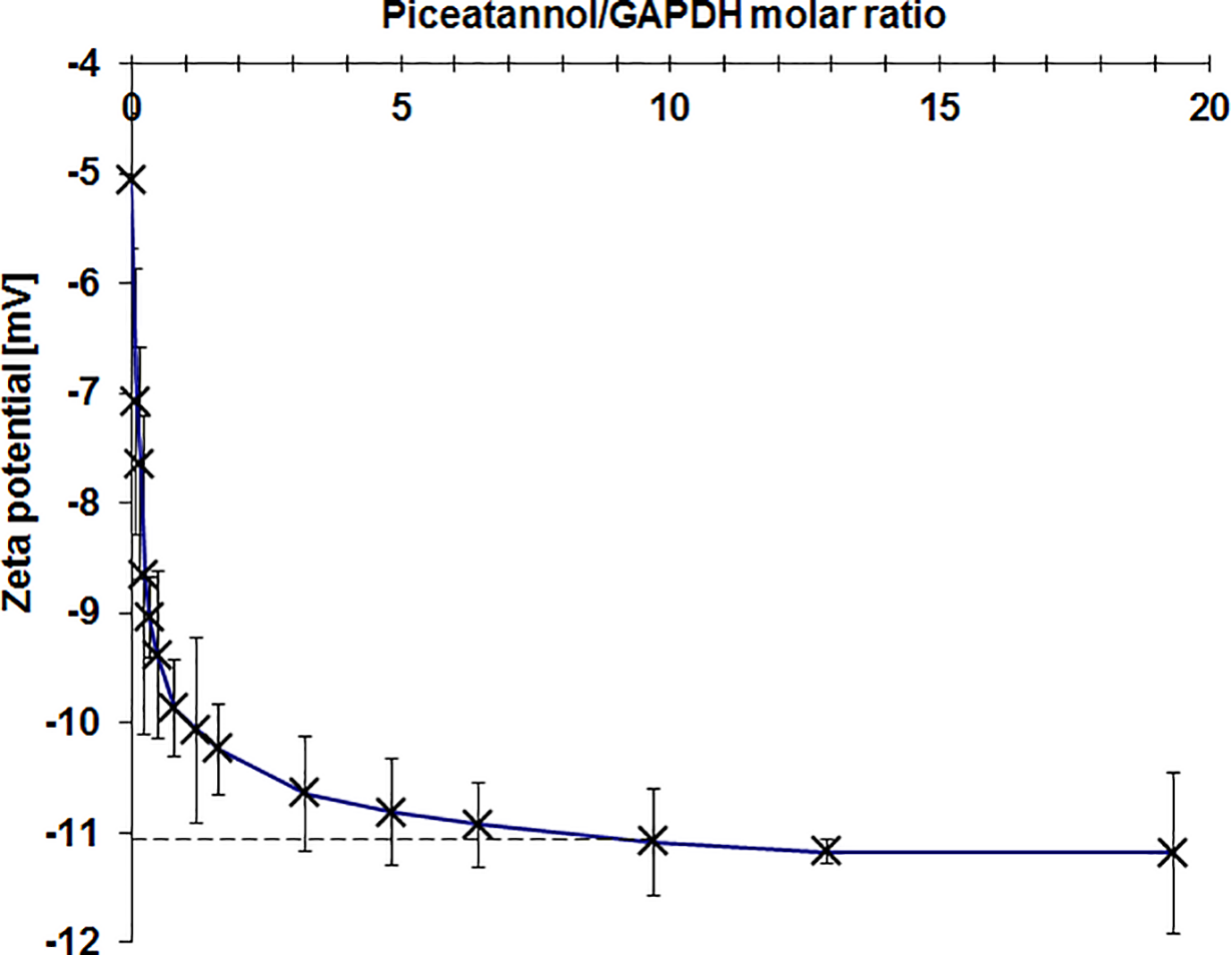
Changes of zeta potential of GAPDH in the presence of piceatannol.

We observed that the zeta potential of GAPDH decreased with increasing molar ratio of the concentration of piceatannol to the dehydrogenase (Fig 5). At a polyphenol-to-protein ratio higher than 8, the potential remained constant. Reduction of potential amounted from -5.1 ± 0.6 mV in the absence of the ligand to -11.2 ± 0.7 mV in its presence. Previous studies indicated slightly smaller changes of charge on the GAPDH surface in the presence of resveratrol (decrease from -4.7mV to -9mV) [8]. The observed differences result from various pKa of both stilbene derivatives which determines the amount of protonated and nonprotonated forms at a specific pH. The pKa values of resveratrol calculated on the basis of proton exchange method equals 9.16 [21]. In the case of piceatannol, the pKa values estimated by the same method vary according to the author: 7.86 and 5.69 calculated by Cordova-Gomez et al. [21] and Lu et al. [31], respectively. Taking into consideration pKa values and physiological pH, 7.4, there have been estimated that in aqueous solution in both cases dominates neutral form. However the proportion of neutral form to anionic form are different. In case of resveratrol only about 1.7% exists in anionic form and 98.3% in neutral form. In case of piceatannol, dependent on established pKa, there have been estimated that in pH 7.4 anionic form occurs in 25.7% [21] or 18.3% [31] and neutral form—74.2% or 81.7%. Based on the Gibbs free energies of deprotonation in water solution established that in case of both compounds the most favourable anions are the products of deprotonation from 4'-OH groups. It was found that the overall reactivity of piceatannol and resveratrol in aqueous solution, at physiological pH, arises almost exclusively from their anionic forms [21].

### Analysis of possible piceatannol binding sites to GAPDH by means of molecular docking

Plausible binding sites of piceatannol to the GAPDH molecule were analyzed using the Ligand Fit algorithm [32]. Based on the conducted analysis detected 48 cavities on the molecular surface of GAPDH that could potentially harbor the studied ligand. Among them the molecular docking method revealed 13 most probable binding sites of piceatannol to the GAPDH tetramer. However after taking into account partially colliding sites, it has been shown that simultaneously 11 molecules of ligand can be bound to dehydrogenase.

More detailed analysis of piceatannol locations in the GAPDH molecule was conducted for the region near the active site of enzyme (near O:Cys-149) and for the region with the highest accessible volume, which is located inside the molecule where the subunits come in contact.

### Analysis of piceatannol interaction in the region located inside the molecule

**Fig 6:**
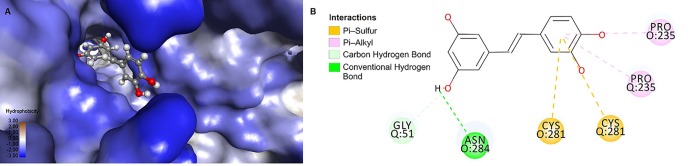
**(A)** The largest accessible region in GAPDH tetramer for piceatannol binding is located inside the molecule, where the subunits come into contact. **(B)** Interactions of piceatannol inside the molecule with amino acid residues from subunit O and Q.

Analysis conducted using the Ligand Fit algorithm detected 10 ligand binding positions with the highest dock-scores (41.2–43.0). The largest accessible region in the GAPDH tetramer, with the highest D-S values was found inside the molecule, where the subunits O and Q come into contact (Fig 6A). Analysis of ligand-protein interactions shows that the identified optimum positions are stabilized by hydrogen bonds (average 3.1 bindings per position) with O:Asn284 (5/10), Q:Gly51 (5/10), Q:Asn284 (5/10) and R:Gln201 (6/10), by hydrophobic interactions (average 1.2/ position) with Q:Pro235 (5/10), Q:Cys281 (4/10) and O:Pro235 (3/10) (Fig 6B and S1 Fig). Among amino acids residues with which ligand can interacts, the most stable interactions are between Cys281 from the subunit O and Q (each 0,8/position). In addition, piceatannol molecule locates inside the GAPDH, much further down the binding cleft as compared to resveratrol (the detailed location of RSV inside the GAPDH tetramer was presented by Rodacka et. al, 2015 [8].

### Analysis of piceatannol locations within the active site, near Cys149

The particular interest have been given to piceatannol poses in the active site of GAPDH, in the region of Cys149 which is involved in the enzymatic reaction. Due to the particular role of this region, a precise analysis of the ligand position was conducted using the CDOCKER algorithm, which is considered to generate a relatively accurate position of the ligand at the binding site [33]. Interactions were characterized by CDOCKER_INTERACTION_ENERGY (-CD_Int_E) values, which describe the overall energy of the ligand−protein interaction (the higher the value, the higher is the interaction energy). Analysis were conducted using the 30 ligand poses with the highest–CDOCKER_INTERACTION_ENERGY values. Analysis was conducted for the active site of the subunit O. The -CD_Int_E values for piceatannol ranged from 28.36 to 33.62 (Table 2).

**Table 2:** Most frequently occurring interactions of piceatannol at the active site of the O subunit of GAPDH. Number in parentheses represents how many times the residue was involved in interaction/30 best poses.

Ligand	CDOCKER_INTERACTION_ENERGY	*hydrogen bonds (H-bonds)*	*hydrophobic interactions*	*interaction of π—sulfur*
*Piceatannol*	28.36–33.62	O:Arg231 (30), O:Ser119 (28), O:Thr150 (22)	O:His176 (26), O:Ala120 (25)	O:Cys149 (14)

Analysis of interactions inside the catalytic center shows that the ligand is stabilized by hydrogen bonds (average 3.97 bindings per position), hydrophobic interactions (average 2.07/pos.) and interaction of π electrons of A and B rings of picetannol with sulfur O:Cys149 (average 0.47/pos.).

**Fig 7:**
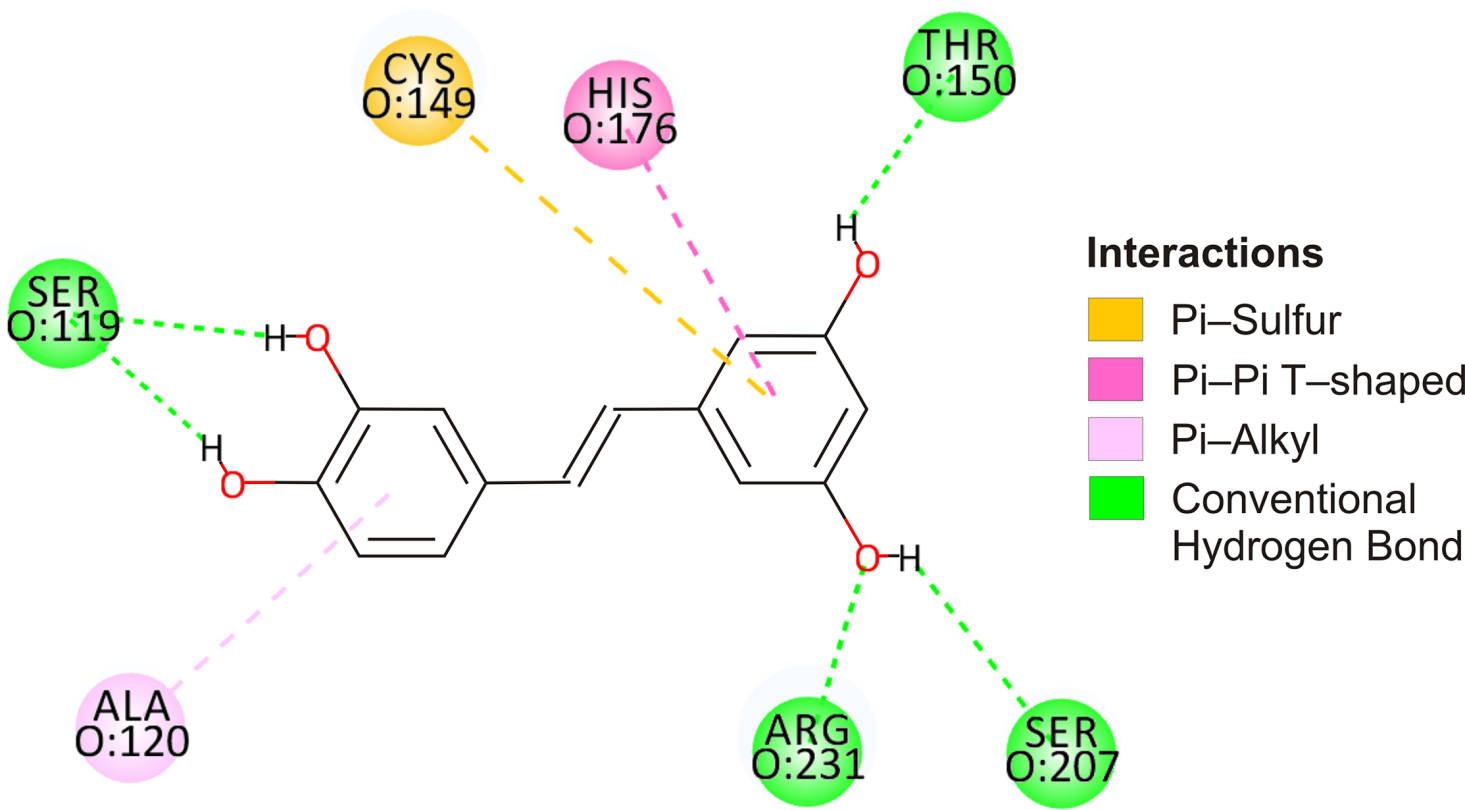
Most frequently interactions of piceatannol with amino acid residues (the catalytic region of enzyme) active site of the O subunit of GAPDH.

The hydrogen bonds (H-bonds) formed by ligand are mainly between O:Arg231 (in 30 analyzed poses), O:Ser119 (28 poses) and O:Thr150 (22 poses). The hydrophobic interactions are mainly between O:His176 (26 interactions) and O:Ala120 (25 interactions) (Table 2). Among 30 of analyzed positions, there found 14 where π electrons of A and B rings of piceatannol interacts with sulfur O:Cys149 (Fig 7).

### The influence of piceatannol on hydrogen peroxide induced GAPDH aggregation

**Fig 8:**
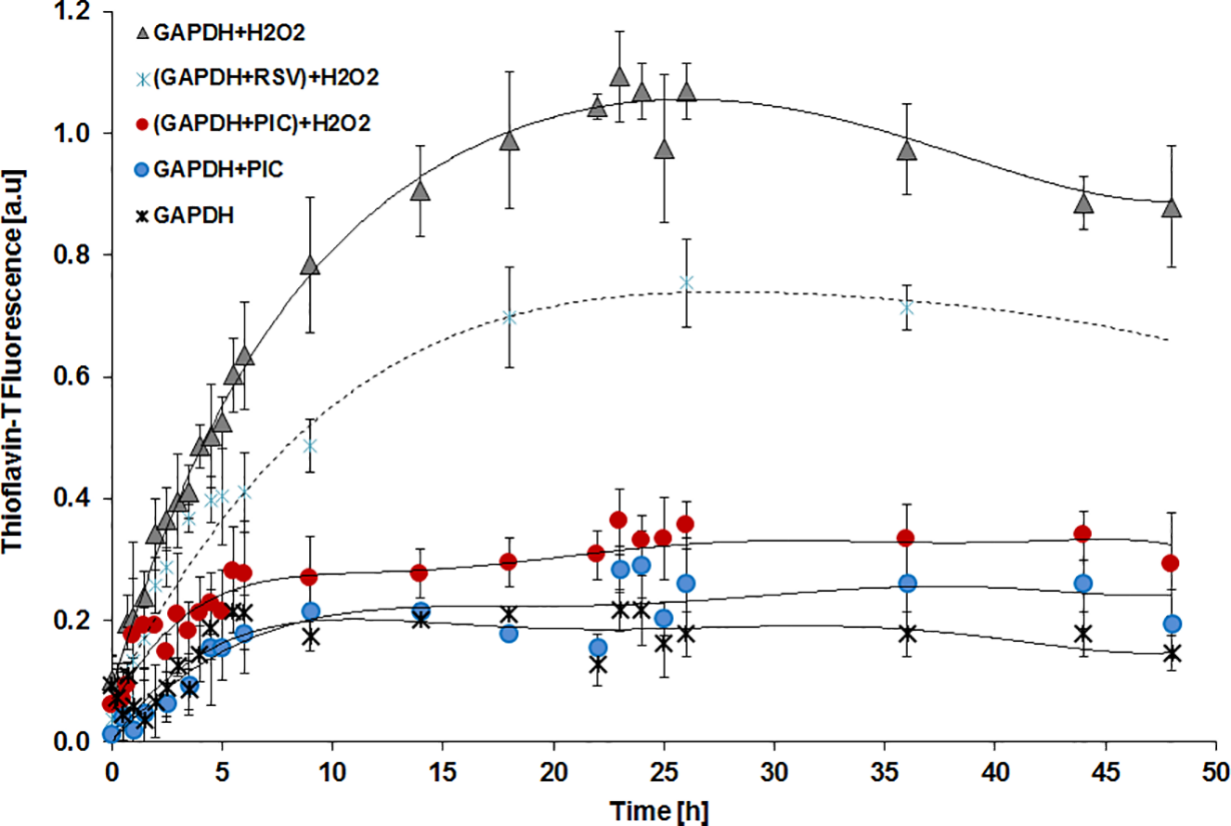
The intensity of thioflavin-T binding-dependent fluorescence of native GAPDH, GAPDH treated with piceatannol (50 μM), GAPDH treated with hydrogen peroxide (3 mM) and GAPDH preincubated for 30 minutes with piceatannol or resveratrol (50 μM) and afterwards treated with H_2_O_2_ at 37°C for the indicated times is shown. Data are means ± SD of n = 4–8 independent measurements.

We subsequently investigated the extent to which piceatannol influences the level of GAPDH aggregation induced by reactive oxygen species (H_2_O_2_). For comparison similar experiments were conducted for resveratrol. We measured the fluorescence intensity of thioflavin-T (ThT) binding, which is one of the hallmarks of amyloid-like fibrils. We observed increased ratio of fluorescence intensity of ThT in GAPDH solution incubated with H_2_O_2_. In samples: native GAPDH, GAPDH incubated with piceatannol or resveratrol (data not shown), the fluorescence of ThT slightly increased during 48 hours of incubation. What’s more in samples where GAPDH was firstly preincubated for 30 minutes with piceatannol, and afterwards with hydrogen peroxide we did not observe any significant aggregation (Fig 8). Resveratrol inhibited aggregation to the lesser extent. The obtained data indicate that piceatannol embedded in the GAPDH molecule and thus protects it against excessive oligomerization and aggregation.

**Fig 9:**
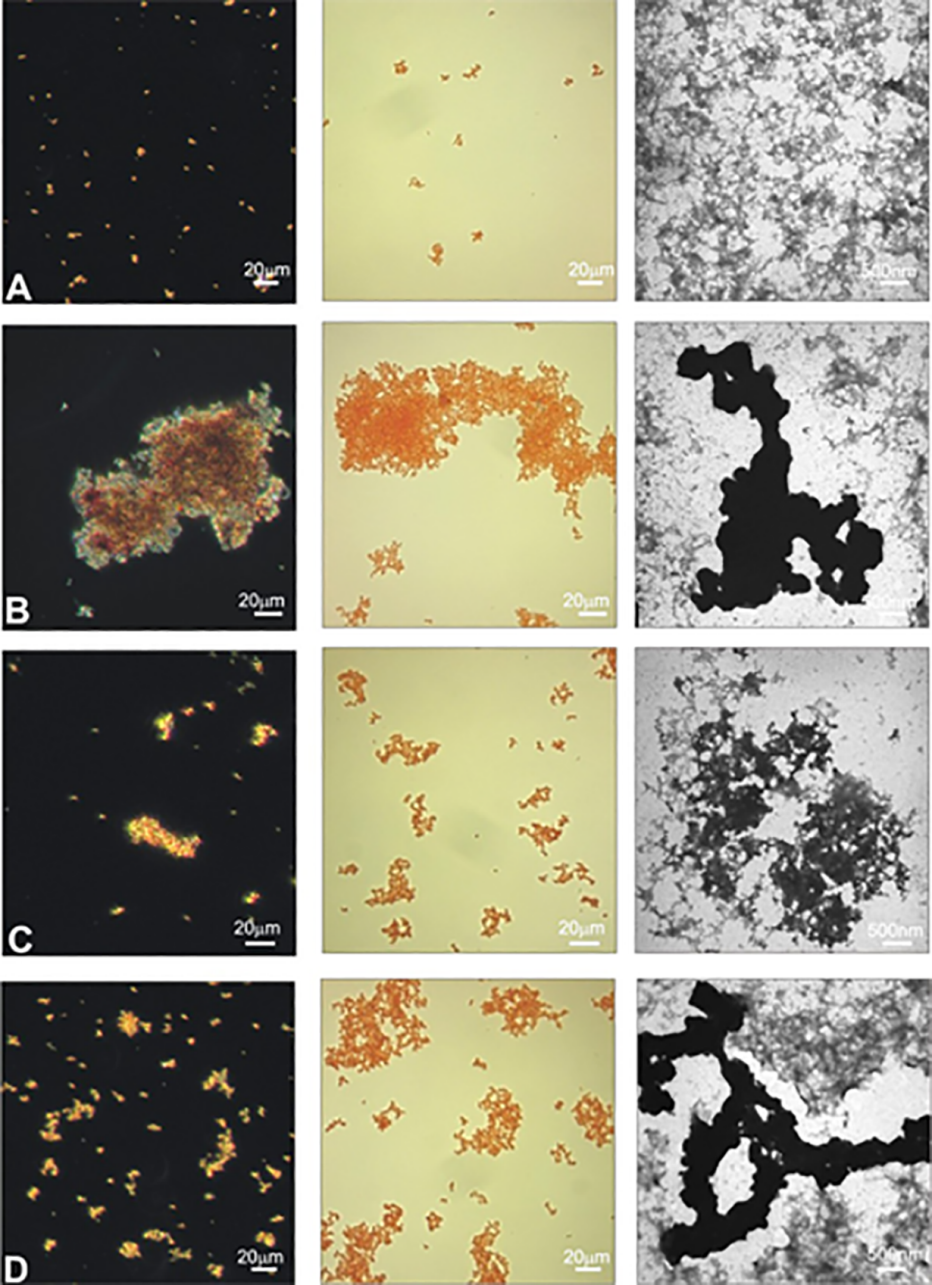
The micrographs of **(A)** GAPDH, **(B)** GAPDH and H_2_O_2_, **(C)** GAPDH with piceatannol and H_2_O_2_, **(D)** GAPDH with resveratrol and H_2_O_2_, from light microscope and transmission electron microscope. Bright field—left panel, dark field—middle panel and electron micrographs-right panel.

Light microscope analysis in both light and dark fields, as well as transmission electron microscopy (TEM) proved that fibrillary structure of GADPH was changing after treatment with H_2_O_2_. 24 hours incubation of GADPH with H_2_O_2_ significantly increased the number of aggregates (Fig 9B). In samples where GAPDH was preincubated with piceatannol (Fig 9C) or resveratrol (Fig 9D) and afterwards exposed H_2_O_2_, the number of aggregates decreased. However, both the size and amount of aggregates were much smaller after the addition of piceatannol then of resveratrol.

## Discussion

The obtained data revealed that piceatannol binds to GAPDH. The ITC analysis indicated that one molecule of the tetrameric enzyme may bind up to 8 molecules of polyphenol (7.3 ± 0.9). The binding process is spontaneous (ΔG<0) and exothermic (ΔH<0), and the formed complex is relatively stable. Changes in the zeta potential were observed as a consequence of piceatannol binding to GAPDH. The zeta potential remained constant when the piceatannol-to-GAPDH ratio was about 7.5. However, the ligand-protein binding did not influence the secondary structure of the enzyme (Fig 4).

Significant inactivation of GAPDH in the presence of piceatannol may primarily result from the binding of the ligand with the GAPDH’s active center, thus blocking the thiol group responsible for the enzymatic activity. Analysis of the ligand-protein interactions near Cys149 indicate that ligand is stabilized by hydrogen bonds (with Arg231, Thr150 and Ser119), hydrophobic interactions (His176, Ala120) and interaction of π electrons of A and B rings of piceatannol with sulfur of Cys149. The above mentioned amino acid residues are directly involved in the catalysis and/or interaction with the substrate and coenzyme, maintaining the corresponding geometry of the active center. Two residues, Cys149 and His176, play a critical role in the catalysis mechanism. Cys149 supplies a sulfhydryl group for nucleophilic attack on G3P, while His176 serves as a base catalyst facilitating a hydride transfer [34,35]. Arg231 and Thr150 are involved in the binding of the phosphate group of the substrate, glyceraldehyde-3-phosphate [35]. Furthermore, the side chain of this arginine has been proposed to play an important role in the regulation of substrate binding and product release [36]. Amino acids residues Ser119 and Ala120 interact with coenzyme NAD^+^. Piceatannol embedded in the active site pocket of GAPDH is likely to be a hindrance and interferes with the binding of the substrate and/or coenzyme, thereby the enzymatic activity of the enzyme is decreasing. In previous studies, we have shown that other stilbene derivatives such as resveratrol and trans-3,3′,5,5′-tetrahydroxy-4′-methoxystilbene (THMS) are incorporated into the GAPDH active center, similar to piceatannol [8]. However, resveratrol interactions are much weaker and involve fewer amino acid residues in this area [8]. As a result, resveratrol did not affect the enzyme activity significantly. On the other hand, THMS binds more strongly in the catalytic pocket than resveratrol. Furthermore, it also interacts with a larger number of amino acid residues (including the formation of hydrogen bonds with Cys149). THMS inactivates GAPDH stronger than resveratrol, but weaker than piceatannol [8].

**Fig 10:**
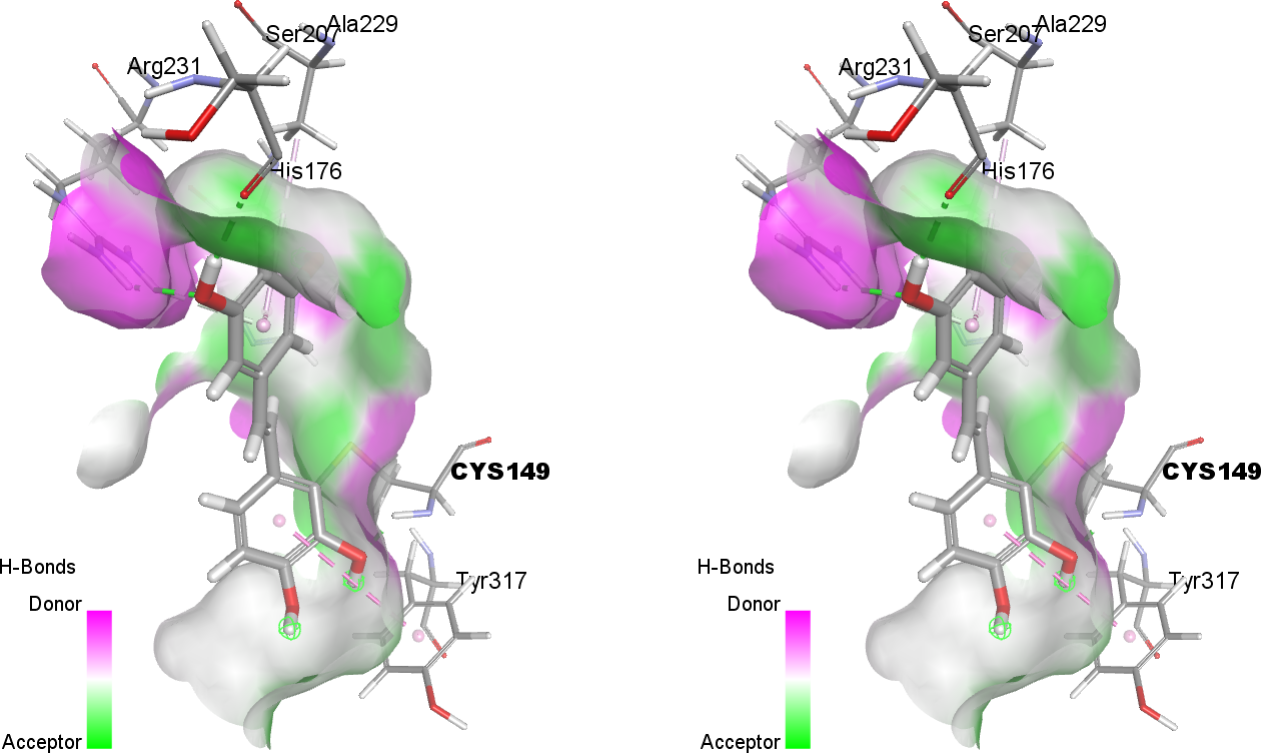
Stereo view of piceatannol bound in GAPDH active center. Covalent binding between nucleophilic thiol group in Cys149 and piceatannol at the 2-C position of the aromatic ring B forms the protein-piceatannol adduct.

**Fig 11:**
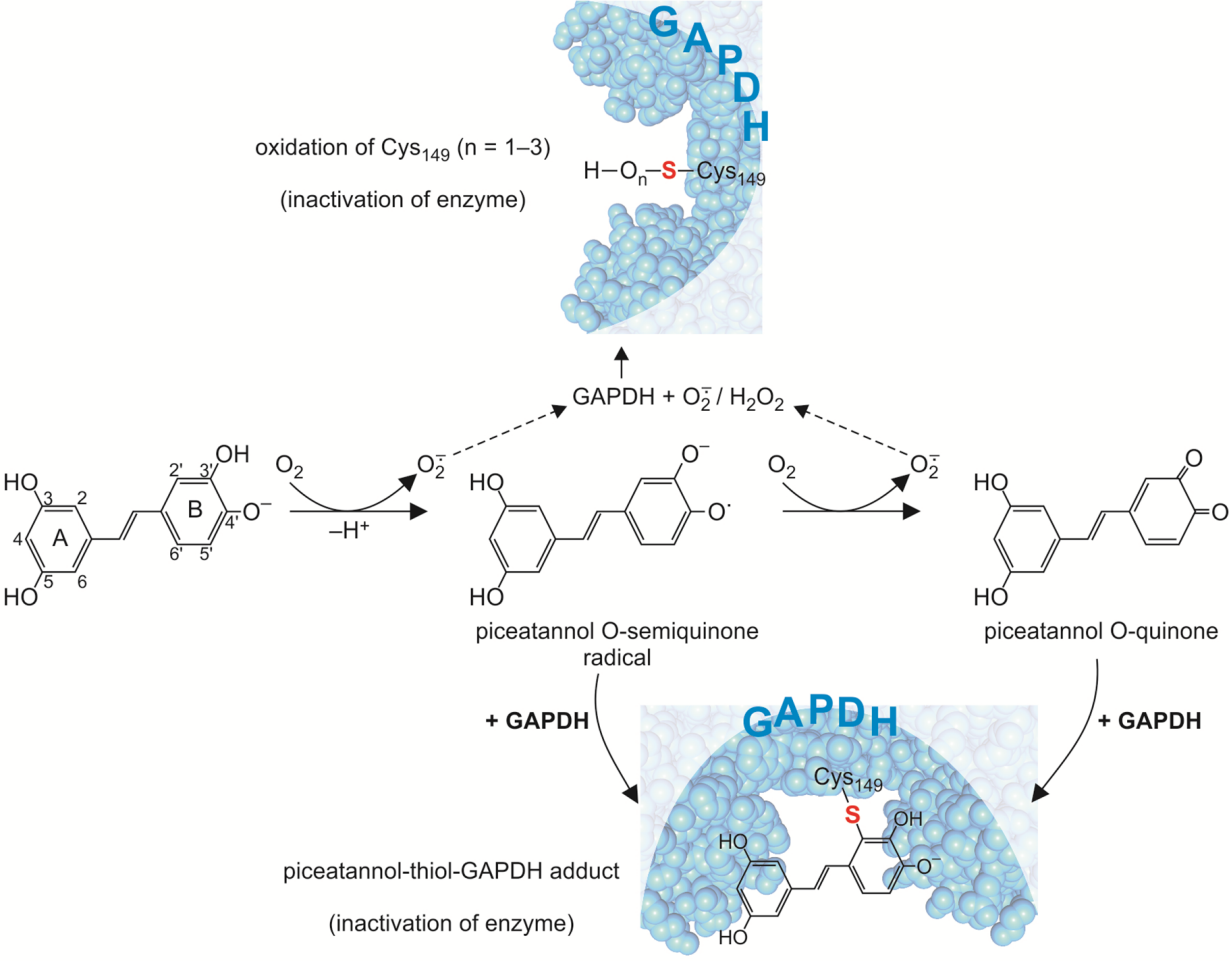
Proposed mechanism for the conjugation of catechol moiety of piceatannol to a nucleophilic thiol group (Cys149) in GAPDH molecule.

It should also be considered that an increased inactivation of GAPDH might be a consequence of assembling an adduct between nucleophilic thiol group in Cys149 and o-semiquinone radical/o-quinone formed during spontaneous oxidation of catechol moiety in piceatannol, in the presence of oxygen (Figs 10 and 11) [37].

The first step of this reaction is one-electron oxidation at position 4’ of catechol group to the *o*-semiquinone radical, during which a superoxide anion radical (O_2_^¯^) is formed. The formed *o*-semiquinone radicals are transient, and they decay rapidly *via* the disproportionation of two radicals to form *o*-quinone and catechol groups. Both, the O-seminquinone and O-quinone spontaneously undergo a nucleophilic attack by the thiol group at the 2’-position of the ring which result in protein-catechol adduct formation [37](Fig 11). Thiols with lower steric hindrance exhibit the highest reactivity [37]. The crystallographic structure shows that the Cys149 residue in GAPDH is located at the bottom of the GAPDH catalytic pocket, in a hydrophilic environment, and is available for reaction with small molecules [9].

Despite the fact that the redox reaction between catechol and oxygen under biological conditions is thermodynamically unfavorable, it could not be excluded. Hydroxyl group at position 4’ in piceatannol at pH 7.4 is deprotonated by approximately 22% [21,31]. It was found that when the catecholic hydroxyl groups is deprotonated, the initial rate of its oxidation increases [37].

Superoxide anion radical formed in one-electron oxidation reaction of catechol moiety, may undergo dismutation to H_2_O_2_. Both superoxide anion and H_2_O_2_ directly oxidize thiol group in the active site of GAPDH, which in consequence may decrease the enzyme catalytic activity [9,10] (Fig 11).

Despite the fact that binding of piceatannol in the dehydrogenase catalytic center and/or the assembly of an adduct with cysteine residue (Cys149) cause a decrease in enzyme activity, it may have beneficial consequences in term of cell’s fate. Takeuchi research group indicated that GAPDH aggregate formation requires oxidation of Cys149 and this process is accelerated by further oxidation of Cys281 [14]. In this study we demonstrated that by binding with GAPDH piceatannol blocks cysteine residue and counteracts its oxidative modifications, that induce oligomerization and GAPDH aggregation. Furthermore, based on the bioinformatic analysis described in the current study, we suggest that cysteine residues (Cys281) of two different subunits may interact directly with piceatannol molecule, which binds in the middle of the enzyme molecule at the interface between two GAPDH subunits. The presence of polyphenol in this region can protect Cys281 from oxidation and consequently, from forming disulfide bridges leading to protein aggregation. This hypothesis is supported by our experimental data. We confirmed by using fluorescence (ThT) as well as microscopy methods (TEM, Congo Red staining) that piceatannol significantly decrease the level of GAPDH aggregation induced by excessive oxidative stress. We observed much weaker protective effect after preincubation of GAPDH with resveratrol. The observed effect occurs probably as a result of much fewer and weaker interactions between resveratrol and amino acids in GAPDH active site [8]. To date there are many evidences which confirm the role of polyphenols in neuroprotection. It has been proven, *inter alia*, that resveratrol decreases the amyloidogenic cleavage of the amyloid precursor protein (APP), enhances clearance of amyloid beta-peptides, and reduces Aβ aggregation [22,38]. Moreover, it’s well known that resveratrol protects neuronal functions through its antioxidant properties. However, there are no data about the influence of stilbene derivatives (piceatannol, resveratrol) on GAPDH aggregation processes. The significance of GAPDH aggregation as the major deadly factor was confirmed both, in Parkinson and Alzheimer model [13,15]. It was also demonstrated that upon exposure to oxidative stress GAPDH binds with Siah and undergoes nuclear translocation, where it induces apoptosis [39,40].

Taking into consideration fact, that neurodegenerative disorders have multiple etiologies, including several pathological signaling pathways occurring successively or simultaneously, therefore it’s important to examine compounds which target different pathological aspects. So far, we had proven that piceatannol promotes neuronal cell survival by attenuation of oxidative stress-mediated cell damage and by sustaining sirtuin 1 (SIRT1), brain derived neurotrophic factor (BDNF), and seladin-1 mRNA on constant level. Furthermore it enhances the activity of glutathione peroxidase (GPx) enzymes as a result of increased GPx expression [17]. Moreover, in this study we have demonstrated that piceatannol notably suppresses aggregation of oxidized GAPDH, and proposed a mechanism which is responsible for this effect. Finally, our latest preliminary studies involving cellular model, suggest that piceatannol targets, *inter alia*, GAPDH in hippocampal cells, which prevents GAPDH from nuclear translocation induced by excessive oxidative stress. In consequence it prevents cells from apoptosis. In summary, these findings provide a new insight into the role of piceatannol interaction with GAPDH and propose a potential therapeutic strategy for some neurological disorders related to GAPDH aggregation.

## Supporting information

S1 Fig Histogram of the most frequent interactions of piceatannol with amino acid residues of GAPDH. (TIF)
