# Supplementary figures and images for: Functional consequences of piceatannol binding to glyceraldehyde-3-phosphate dehydrogenase
Source: PLoS One. 2018 Jan 3;13(1):e0190656. doi: 10.1371/journal.pone.0190656 (PMC5752021; doi:10.1371/journal.pone.0190656)

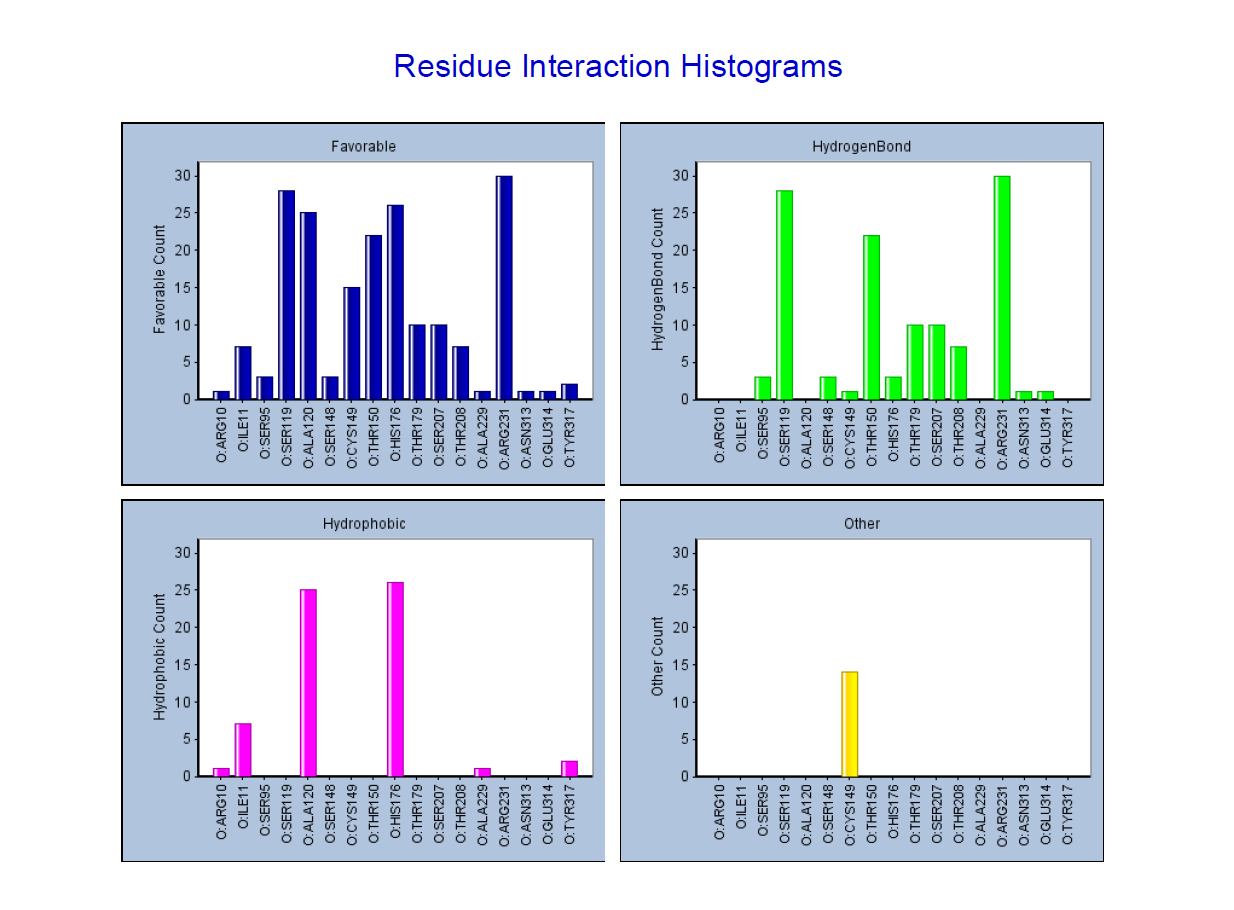

Supplement: S1 Fig — (TIF) [file pone.0190656.s001.tif]
